# A Simple Approach to Achieve Modified Projective Synchronization between Two Different Chaotic Systems

**DOI:** 10.1155/2013/568194

**Published:** 2013-09-25

**Authors:** Lei Wang, Bin Zhen, Jian Xu

**Affiliations:** ^1^College of Hydraulic and Environmental Engineering, China Three Gorges University, Yichang 443002, China; ^2^School of Aerospace and Mechanics Engineering, Tongji University, Shanghai 200092, China

## Abstract

A new approach, the projective system approach, is proposed to realize modified projective synchronization between two different chaotic systems. By simple analysis of trajectories in the phase space, a projective system of the original chaotic systems is obtained to replace the errors system to judge the occurrence of modified projective synchronization. Theoretical analysis and numerical simulations show that, although the projective system may not be unique, modified projective synchronization can be achieved provided that the origin of any of projective systems is asymptotically stable. Furthermore, an example is presented to illustrate that even a necessary and sufficient condition for modified projective synchronization can be derived by using the projective system approach.

## 1. Introduction

Chaos synchronization has been studied with increasing interest over the last few decades due to its numerous potential applications [1–3]. Different types of chaos synchronization behaviors have been observed and investigated, such as complete synchronization [3], phase synchronization [4], antiphase synchronization [5], lag synchronization [6], generalized synchronization [7], and projective synchronization [8–15]. Among all these types of chaos synchronization, projective synchronization received many attentions in recent years because of its association with projective synchronization and generalized one. In addition, projective synchronization was used to extend binary digital to M-nary digital communication for achieving fastness and security.

Projective synchronization was first reported by Mainieri and Rehacek [12] in a class of systems with partial linearity in which drive and response vectors evolve on a proportional scale. Xu and Li [16], Wen and Xu [17], and Yan and Li [18] extended the projective synchronization feature to general nonlinear systems including nonpartially linear chaotic systems by applying controllers to response systems, which is called generalized projective synchronization. This synchronization has the same topological invariants as those of projective synchronization. Complete synchronization and antisynchronization are special cases of generalized projective synchronization. Recently, a new type of projective synchronization, modified projective synchronization, was considered by Li [10, 11] in which the response of synchronized dynamical states can synchronize up to a constant matrix. Modified projective synchronization was an extension of generalized projective synchronization. In most previous research works, controllers added to the response system to achieve projective synchronization were designed by Lyapunov stability theory, and therefore most proposed schemes were specific. Additionally, the added controllers were sometimes too complex to realize physically. Thus, a more simple and available controller to achieve projective synchronization between two different chaotic systems is desirable to find.

In this paper, “the projective system approach” is proposed to achieve modified projective synchronization between two different chaotic systems. By simple analysis of trajectories in the phase space, a projective system of the two original chaotic systems is obtained to replace the errors system to judge the occurrence of modified projective synchronization. Then modified projective synchronization between two chaotic systems can be realized if the states of the projective system are asymptotically stable at the origin. A simple synchronization criterion based on the projective system approach is derived independent of finding Lyapunov function. An example is given to show that even a necessary and sufficient condition for modified projective synchronization can be found by using the criterion.

The rest of the paper is organized as follows. In Section 2, modified projective synchronization of two different chaotic systems is theoretically analyzed. A simple criterion for realizing modified projective synchronization is obtained by using the proposed projective system approach. In Section 3, an example is given to numerically demonstrate the effectiveness of the proposed approach. In Section 4, another example is provided to verify the effectiveness of the proposed approach by comparing the results obtained by the projective system approach with those obtained by Lyapunov method. Finally, conclusions are drawn in Section 5.

## 2. Modified Projective Synchronization of Two Chaotic Systems

 The two chaotic (drive and response) systems can be given in the following form:

(1) x˙=f(x),y˙=g(y)+u,

where *x*, *y* ∈ *R* ^*n*^, *f*, *g* are continuous vector functions and *u* is the controller to be designed. If there exists a constant matrix *α* = diag⁡(*α*_1_, *α*_2_,…, *α*_*n*_), such that lim_*t*→+*∞*_||*y* − *αx*|| = 0, then the two chaotic systems are said to be modified projective synchronization, and *α* is a scaling matrix [10, 11]. Obviously, complete synchronization and projective synchronization are the special cases of modified projective synchronization where *α*_1_ = *α*_2_ = ⋯ = *α*_*n*_ = 1 and *α*_1_ = *α*_2_ = ⋯ = *α*_*n*_, respectively.

Consider that the controller *u* in system (1) is designed as

(2) $$\begin{array}{c} u=\alpha f\left(x\right)-g\left(\alpha x\right)+k\left(y-\alpha x\right), \end{array}$$

where *k* = diag⁡(*k*_1_, *k*_2_,…, *k*_*n*_). The synchronization errors between the drive and response systems are defined as

(3) $$\begin{array}{c} e=y-\alpha x; \end{array}$$

then system (1) can be written as

(4) $$\begin{array}{c} \dot{x}=f\left(x\right),\\ \dot{e}=g\left(e+\alpha x\right)-g\left(\alpha x\right)+ke. \end{array}$$

**Figure 1:**
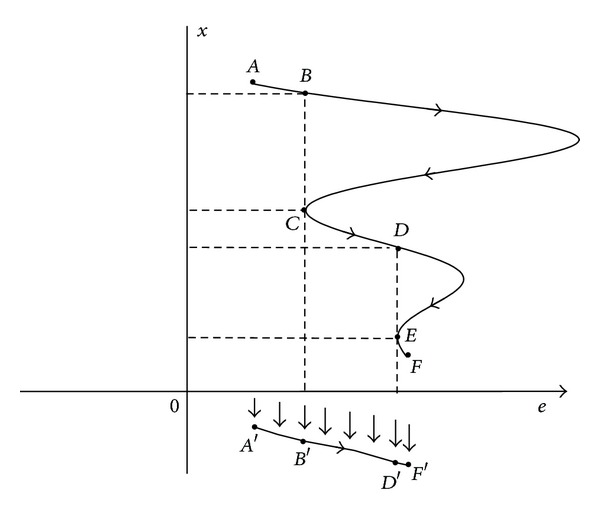
Analysis of trajectories in the phase space of system (4). The trajectory from point *A* to point *F* is called $\overset{⌢}{AF}$. Points *B*, *D* and points *C*, *E* have identical *e* values, respectively. Trajectories $\overset{⌢}{AB}$, $\overset{⌢}{CD}$, and $\overset{⌢}{EF}$ are called $\overset{⌢}{A\text{–}F}$, which are discontinuous at points *B*, *D*. $\overset{⌢}{A^{\prime }F^{\prime }}$ represents a smooth trajectory from point *A*′ to point *F*′.

Consider the phase space of system (4) presented in Figure 1; a trajectory starting from point *A* moves to point *F* (called $\overset{⌢}{AF}$). Assume that points *B*, *D* and points *C*, *E* have identical *e* values, respectively. The question that we need to address is as follows: under what condition does trajectory $\overset{⌢}{AF}$ approach *x*-axis infinitely? Then, trajectories $\overset{⌢}{BC}$ and $\overset{⌢}{DE}$ do not require to be taken into consideration since the distance from point *B* (*D*) to *x*-axis is equal to that from point *C* (*E*) to *x*-axis. We can investigate trajectories $\overset{⌢}{AB}$, $\overset{⌢}{CD}$, and $\overset{⌢}{EF}$ (called $\overset{⌢}{A\text{–}F}$) instead of trajectory $\overset{⌢}{AF}$ to determine whether *e* → 0 more directly. From Figure 1, it is clear that trajectory $\overset{⌢}{A\text{–}F}$ can be obtained through regarding points on trajectory $\overset{⌢}{AF}$ with identical *e* value as one point.

It should be noted that $\overset{⌢}{A\text{–}F}$ is discontinuous at points *B*, *D* in the direction of *x*-axis. However, only the evolution of *e* values of points on $\overset{⌢}{A\text{–}F}$ is of interest to our study. Then consider all the points on $\overset{⌢}{A\text{–}F}$ are translated along *x*-axis to form a smooth curve $\overset{⌢}{A^{\prime }F^{\prime }}$, which is equivalent to curve $\overset{⌢}{AF}$ to our subject (Figure 1). According to the analysis above, $\overset{⌢}{A^{\prime }F^{\prime }}$ can be obtained by letting *x* = *h*(*e*) (*h* is smooth enough) in system (4):

(5) $$\begin{array}{c} \frac{\partial h\left(e\right)}{\partial t}=f\left(h\left(e\right)\right),\\ \dot{e}=g\left(e+\alpha h\left(e\right)\right)-g\left(\alpha h\left(e\right)\right)+ke. \end{array}$$

System (5) is called the projective systemof system (4). From the analysis of trajectories in the phase space of system (4), the two systems in (1) can achieve modified projective synchronization provided that *e* → 0 holds in system (4). Then, projective system (5) can be used to replace system (4) to judge the occurrence of modified projective synchronization.

For a sufficiently small *e*, the right hand of equation *x* = *h*(*e*) can be expanded as

(6) $$\begin{array}{c} x=h\left(e\right)=h_{0}+\frac{\partial h\left(0\right)}{\partial e}e+O_{1}\left(e\right), \end{array}$$

where *h*_0_ = *h*(0), *O*_1_(*e*) represents the higher order terms of *e*. Substituting (6) into the first equation in system (5) yields

(7) $$\begin{array}{c} f\left(h_{0}\right)=0. \end{array}$$

*h*_0_ can be derived by solving (7). The second equation of system (5) can be approximated by

(8) e˙=g(e+αh(e))−g(αh(e))+ke,=(∂g(z)∂z|z=αh0+k)e+O2(e),

where *O*_2_(*e*) represents the higher order terms of *e*. It is clear that *e* → 0 holds in system (5), also in system (4), if the matrix

(9) $$\begin{array}{c} P\left(h_{0}\right)={\frac{\partial g(z)}{\partial z}|}_{z=\alpha h_{0}}+k \end{array}$$

is stable. That is, modified projective synchronization between two different chaotic systems in (1) is achieved. The approach introduced in this section to realize modified projective synchronization between two different chaotic systems can be called the projective system approach. This approach has been successfully applied to investigate the generalized synchronization in unidirectionally coupled systems in [19].

It should be pointed out that the projective system of system (4) may not be unique because function *h*(*e*) may not be unique. Furthermore, the possible number of the projective systems of system (4) depends on the number of real roots of (7). From Figure 1, modified projective synchronization occurs as long as there exist trajectories approaching *x*-axis. Assuming that *h*_01_, *h*_02_,…, *h*_0*n*_ are *n* real roots of (7), then modified projective synchronization appears if any matrix *P*(*h*_0*i*_), 1 ≤ *i* ≤ *n*, is stable. In this sense, more equilibria possessed by the drive system mean a higher chance of modified projective synchronization in system (1).

Clearly, the projective system approach introduced in this paper works provided that the drive system in (1) possesses equilibria. For the physical systems in the real world, such condition is very easy to be satisfied. Thus, the projective system approach can be widely used.

## 3. A Numerical Example of Modified Projective Synchronization

In the section, an example is given to numerically demonstrate the validity of the projective system approach. Consider the Lorenz system as the drive system

(10) x˙1=σ(x2−x1),x˙2=γx1−x1x3−x2,x˙3=x1x2−βx3,

where *σ* = 10, *γ* = 28, and *β* = 8/3. The Chen system [20] is adopted as the response system, which is defined as

**Figure 2:**
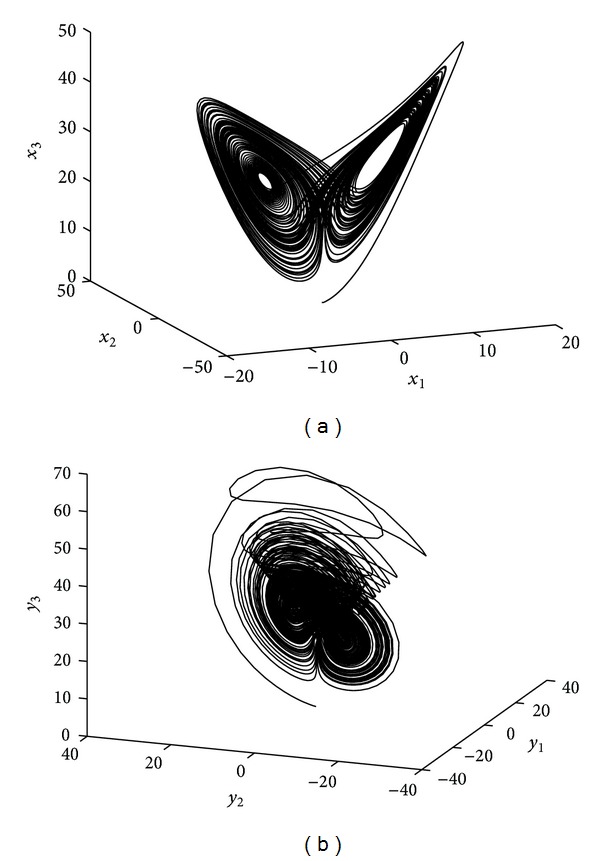
The chaotic attractors of system (10) and system (11) without the control. (a) The chaotic attractor of Lorenz system (10). (b) The chaotic attractor of Chen system (11) without the control.

(11) y˙1=a(y2−y1)+u1,y˙2=(c−a)y1−y1y3+cy2+u2,y˙3=y1y2−by3+u3,

where *a* = 35, *b* = 3, *c* = 28, and *u* = (*u*_1_, *u*_2_, *u*_3_)^*T*^ is the controller. The chaotic attractors of system (10) and system (11) without the controller are shown in Figures 2(a) and 2(b), respectively.

The controller *u* is designed according to (2) as

(12) u1=(a−σ)α1x1+(σα1−aα2)x2+k(y1−α1x1),u2=[γα2−(c−a)α1]x1+(α1α3−α2)x1x3−(c+1)α2x2+k(y2−α2x2),u3=(α3−α1α2)x1x2+(b−β)α3x3+k(y3−α3x3),

where *α* = diag⁡(*α*_1_, *α*_2_, *α*_3_) is the scaling matrix, *k* is the control parameter.

From (7), one has

(13) h01=[0,0,0]T,h02=[62,62,27]T,h03=[−62,−62,27]T.

From (9), the discriminant matrix for modified projective synchronization between systems (10) and (11) can be expressed by

(14)   P(h01)=[−a+ka0c−ac+k000−b+k],P(h02,h03)=[−a+ka0c−a−27α3c+k∓6α12±6α22±6α12−b+k].

**Figure 3:**
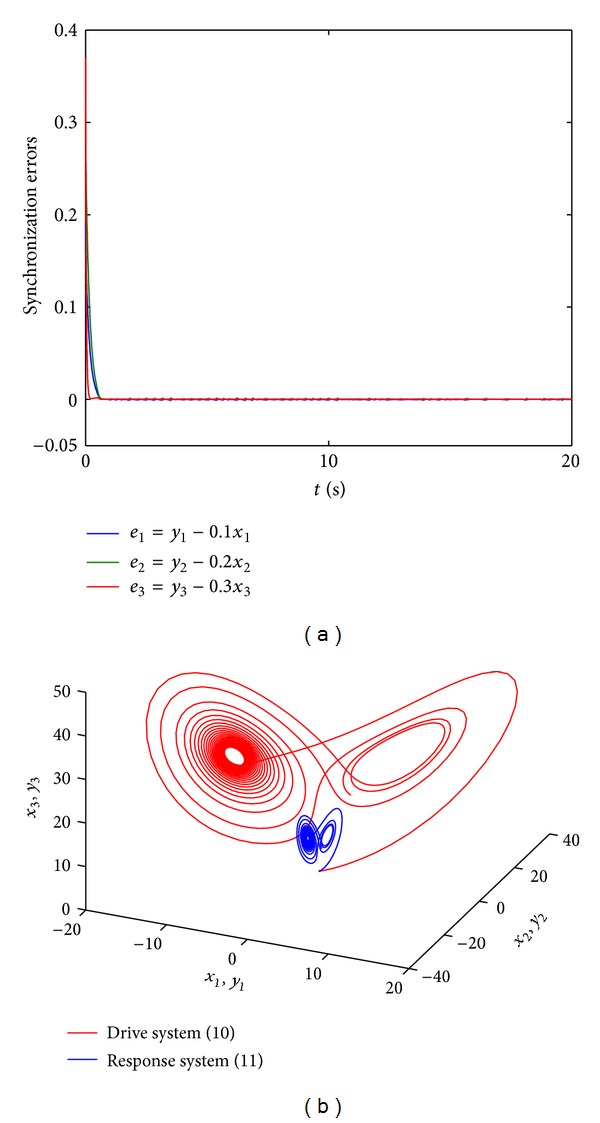
Modified projective synchronization between systems (10) and (11) can be realized for the scaling matrix *α* = diag⁡(0.1,0.2,0.3) when *k* = −30. The initial values of the drive and response systems are chosen as (*x*_1_(0), *x*_2_(0), *x*_3_(0)) = (0.1,0.1,0.1) and (*y*_1_(0), *y*_2_(0), *y*_3_(0)) = (0.2,0.3,0.4), respectively. (a) The synchronization errors between systems (10) and (11). (b) The chaotic attractors of system (10) and system (11) with the control.

**Figure 4:**
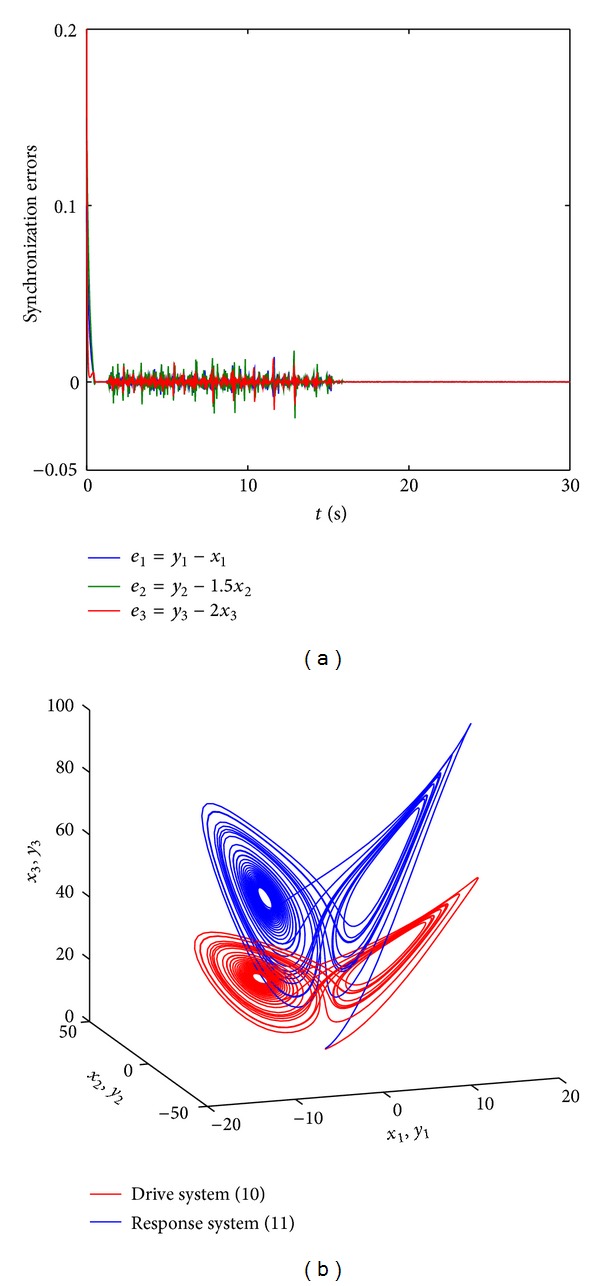
Modified projective synchronization between systems (10) and (11) can be realized for the scaling matrix *α* = diag⁡(1,1.5,2) when *k* = −30. The initial values of the drive and response systems are chosen as (*x*_1_(0), *x*_2_(0), *x*_3_(0)) = (0.1,0.1,0.1) and (*y*_1_(0), *y*_2_(0), *y*_3_(0)) = (0.2,0.3,0.4), respectively. (a) The synchronization errors between systems (10) and (11). (b) The chaotic attractors of system (10) and system (11) with the control.

All the eigenvalues of matrix *P*(*h*_01_) have negative real parts provided that $k<(1/2)(a-c-\sqrt{c^{2}+6ac-3a^{2}})=-23.84$. It is important to point out that this condition for the control parameter *k* can guarantee the occurrence of modified projective synchronization between systems (10) and (11) for any given scaling matrix *α* = diag⁡(*α*_1_, *α*_2_, *α*_3_). The theoretical result is illustrated by numerical calculation results presented in Figures 3 and 4. In the numerical simulations (Figures 3 and 4) the control parameter *k* equals −30 and the initial values of the drive and response systems are chosen as (*x*_1_(0), *x*_2_(0), *x*_3_(0)) = (0.1,0.1,0.2) and (*y*_1_(0), *y*_2_(0), *y*_3_(0)) = (0.2, 0.3, 0.4), respectively. The scaling matrix is taken as *α* = diag⁡(0.1,0.2,0.3) and *α* = diag⁡(1,1.5,2) in Figures 3 and 4, respectively.

**Figure 5:**
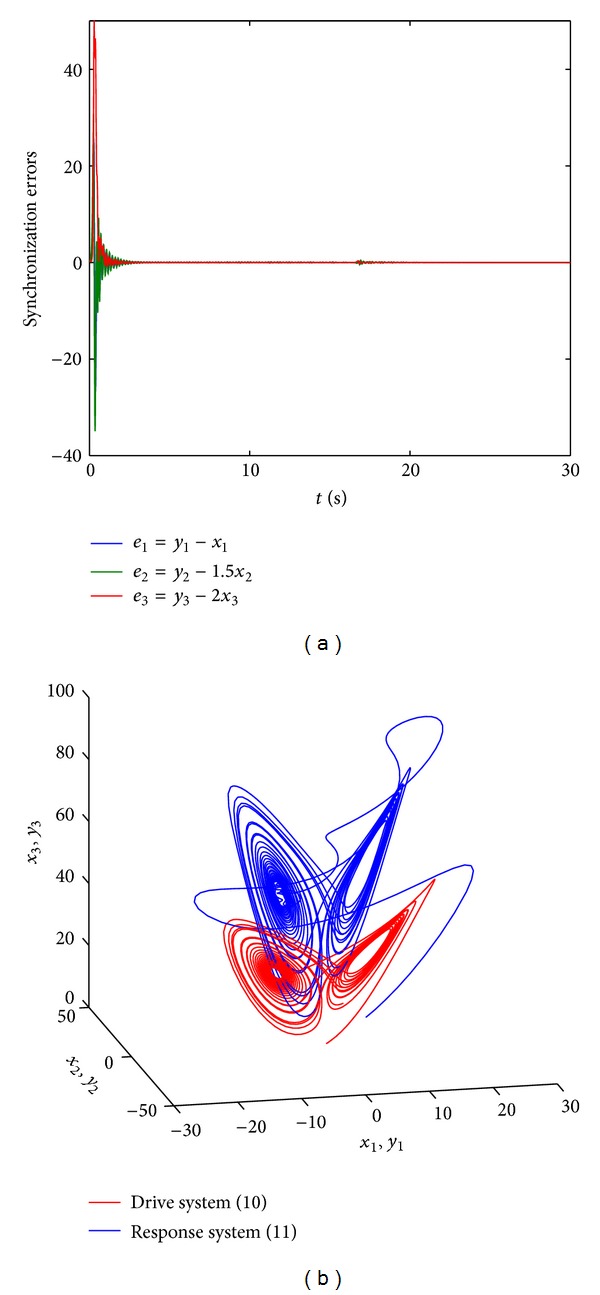
Modified projective synchronization between systems (10) and (11) can be realized for the scaling matrix *α* = diag⁡(1,1.5,2) when *k* = −0.5. The initial values of the drive and response systems are chosen as (*x*_1_(0), *x*_2_(0), *x*_3_(0)) = (0.1,0.1,0.1) and (*y*_1_(0), *y*_2_(0), *y*_3_(0)) = (0.2,0.3,0.4), respectively. (a) The synchronization errors between systems (10) and (11). (b) The chaotic attractors of system (10) and system (11) with the control.

According to the analysis in the previous section, the condition for *k* derived based on *P*(*h*_01_) is not necessary to realized modified projective synchronization between systems (10) and (11). In fact, modified projective synchronization occurs as long as all the eigenvalues of matrix *P*(*h*_01_) or *P*(*h*_02_) or *P*(*h*_03_) have negative real parts. If the scaling matrix is taken as *α* = diag⁡(*α*_1_, *α*_2_, *α*_3_) = diag⁡(0.1,0.2,0.3) and the control parameter *k* satisfies *k* < 1.04, matrix *P*(*h*_02_) or *P*(*h*_03_) has no eigenvalue with nonnegative real parts. Then, modified projective synchronization between systems (10) and (11) still can be achieved for *α* = diag⁡(0.1,0.2,0.3) when *k* > −30. The numerical results are shown in Figure 5, in which *α* = diag⁡(0.1,0.2,0.3), *k* = −0.5, and the initial values of the drive and response systems are still taken as (*x*_1_(0), *x*_2_(0), *x*_3_(0)) = (0.1,0.1,0.2) and (*y*_1_(0), *y*_2_(0), *y*_3_(0)) = (0.2,0.3,0.4), respectively.

## 4. Discussion

In this section, another example is provided to compare the results obtained by the projective system approach with those obtained by Lyapunov method. Consider the following coupled Lorenz systems

(15) x˙1=−μx1+μx2,x˙2=(ξ−x3)x1−x2,x˙3=x1x2−ρx3,y˙1=−μy1+μy2,y˙2=(ξ−x3)y1−y2,

where *μ*, *ρ*, and *ξ* are system parameters. Two-variable partially projective synchronization has been found in system (15) [21]. That is, lim⁡_*t*→*∞*_||*y*_1,2_ − *αx*_1,2_|| = 0 holds under certain conditions, in which *α* ∈ *R* is the scaling factor. Next, the synchronization conditions for system (15) are separately derived by Lyapunov method and the projective system approach.

According to [21], lim⁡_*t*→*∞*_||*y*_1,2_ − *αx*_1,2_|| = 0 is equivalent to lim⁡_*t*→*∞*_(*x*_1_ *y*_2_ − *y*_1_ *x*_2_) = 0. Denote the error vector by *e* = *x*_1_ *y*_2_ − *y*_1_ *x*_2_; then error system can be written as

(16) $$\begin{array}{c} \dot{e}={\dot{x}}_{1}y_{2}+x_{1}{\dot{y}}_{2}-{\dot{y}}_{1}x_{2}-y_{1}{\dot{x}}_{2}. \end{array}$$

Lyapunov function is chosen as *V*(*t*) = (1/2)*e* ^2^; then

(17) $$\begin{array}{c} \dot{V}=e\dot{e}=e\left({\dot{x}}_{1}y_{2}+x_{1}{\dot{y}}_{2}-{\dot{y}}_{1}x_{2}-y_{1}{\dot{x}}_{2}\right)=-\left(\mu +1\right)e^{2}. \end{array}$$

Obviously, $\dot{V}<0$ as long as *μ* > −1, which is the condition for two-variable partially projective synchronization in system (15). It is worth pointing out that condition *μ* > −1 also is necessary for the occurrence of the projective synchronization since $\dot{V}>0$ if *μ* < −1. However, it is generally difficult to find such proper Lyapunov function for any two coupled chaotic systems.

In the following, the projective system approach is applied to get the condition for synchronization. Comparing system (1) with system (15), the controller *u* can be expressed by

(18) u1=0,u2=−y1x3.

From (7), one has

(19) h01=[0,0,0]T,h02=[±ρ(ξ−1),±ρ(ξ−1),ξ−1]T.

From (9), the discriminant matrix for synchronization can be given by

(20) $$\begin{array}{c} P\left(h_{01}\right)=\left[\begin{array}{cc} -\mu & \mu \\ \xi & -1 \end{array}\right],\;\;P\left(h_{02},h_{03}\right)=\left[\begin{array}{cc} -\mu & \mu \\ 1 & -1 \end{array}\right]. \end{array}$$

According to the projective system approach, two-variable partially projective synchronization occurs in system (15) provided that any of matrices *P*(*h*_01_) and *P*(*h*_02_, *h*_03_) is stable. The condition for synchronization also is *μ* > −1 based on the projective system approach, which shows that a necessary and sufficient condition for modified projective synchronization may be found by using the approach.

## 5. Conclusion

In this paper, the projective system approach is proposed to realize modified projective synchronization of two different chaotic systems up to a desired scaling matrix. It is found that a projective system can be obtained from the original system to judge the occurrence of modified projective synchronization. A numerical example is given to illustrate the effectiveness of the projective system approach. Furthermore, another example of two-variable partially projective synchronization in two coupled Lorenz systems shows that a necessary and sufficient synchronization condition can be derived by using the projective system approach. Theoretical analysis and numerical simulations demonstrate that, although the projective system may be not unique, modified projective synchronization between two different chaotic systems can be achieved provided that the origin of any of projective systems is asymptotically stable.
